# Age rather than diabetes duration predicts the severity of sensory neuropathy in type 2 diabetes mellitus

**DOI:** 10.3389/fendo.2025.1763487

**Published:** 2026-01-23

**Authors:** Áron T. Kiss, Árpád Kézdi, Magdolna Békeffy, Csaba G Koós, Anna E. Körei, Adrienn Menyhárt, Dóra M. Balogh, Péter Kempler, Ádám Gy. Tabák, Viktor J. Horváth

**Affiliations:** 1 Department of Medicine and Oncology, Semmelweis University, Budapest, Hungary; 2 Biatorbágy City Health Care Nonprofit Ltd. Specialist Clinic, Biatorbágy, Hungary; 3 Department of Public Health, Semmelweis University, Budapest, Hungary

**Keywords:** age, peripheral neuropathy, sensory test, symptoms, type 2 diabetes

## Abstract

**Introduction:**

In patients with type 2 diabetes, factors beyond hyperglycemia may contribute to the development of sensory neuropathy. This study aimed to determine whether diabetes duration was the strongest predictor of prevalent sensory neuropathy and to compare the diagnostic performance of commonly used clinical screening methods.

**Materials and methods:**

A total of 711 patients with type 2 diabetes from the Budapest metropolitan area were assessed between 2016 and 2022 using routine sensory neuropathy screening tests (128-Hz tuning fork and 10-g monofilament). A brief standardized questionnaire was also administered to support neuropathy detection. Independent predictors of sensory neuropathy were identified using binary logistic regression models and interrater agreement between diagnostic methods was assessed with Cohen’s kappa.

**Results:**

Agreement between diagnostic methods was only moderate (Cohen’s kappa: 0.3–0.4). In the final adjusted models, age emerged as the only independent predictor of neuropathy detected by tuning fork testing (OR 1.04; 95% CI 1.02–1.06), whereas for the monofilament test, self-reported neuropathic symptoms remained independently associated. Consistent with this, positive responses to standardized symptom questions correlated with both diagnostic tests, supporting their clinical validity.

**Conclusion:**

The overlap between routinely applied clinical methods for detecting sensory neuropathy is limited. Our findings suggest that, in an aging population with type 2 diabetes, peripheral nerve damage identified by standard screening tools is more strongly associated with age than with diabetes duration when assessed by commonly used bedside screening tools. Prospective studies are warranted to better differentiate diabetic neuropathy from age-related nerve dysfunction.

## Introduction

Distal symmetric peripheral neuropathy (DSPN) is a common complication of both type 1 and type 2 diabetes, although other conditions may also contribute to its development. In general population, the prevalence of peripheral neuropathy ranges from 2% to 12%, with higher rates observed in individuals over 50 years of age (1–3). DSPN is of idiopathic origin in 25% to 46% of cases, with the prevalence of idiopathic forms increasing with age (4). In contrast, among patients with diabetes, the prevalence of DSPN ranges from 13% to 50%, depending on age, diabetes type and duration, diagnostic method, level of glycemic control (2, 5) and other factors.

While diabetes is a major risk factor for DSPN, other contributors include nerve compression or injury, alcohol use, chemotherapeutic agents, dialysis, and nutritional deficiencies (6, 7). Regardless of the etiology, clinical manifestations of DSPN are similar and typically include sensory loss, numbness, burning sensations, or pain. However, certain features - such as the “stocking-and-glove” distribution of symptoms - are considered more characteristic for diabetes-associated DSPN, but up to 50% of diabetic peripheral neuropathy may be asymptomatic (8). It is important to note that clinically significant nerve damage usually results from chronic exposures to multiple pathogenic factors, and thus the precise etiology of DSPN cannot always be determined at the time of clinical evaluation. As a consequence, the diagnosis often reflects a “final common pathway” of neuropathic injury rather than a disease-specific mechanism.

Beyond peripheral sensory pathways, autonomic measures also show pronounced age dependence in healthy people. In classic normative studies, heart-rate reflex indices (e.g., deep-breathing and postural ratios) and overall heart rate variability declined steadily with age, prompting age-stratified normal ranges for tests of cardiac vagal function (9, 10). These data underscore that routine neuro-cardiovascular and somatosensory tests can both be influenced by physiological ageing, independent of diabetes. Importantly, vibration perception thresholds are known to be strongly age-dependent even in healthy individuals, as demonstrated in different studies (11, 12). The relative contribution of different factors to the development of DSPN is still discussed, and designing human studies that can disentangle the effects of coexisting risk factors is particularly challenging. Consequently, retrospective studies remain important to assess the relative weight of different influences in DSPN development.

In this study, we analyzed data from a suburban diabetes outpatient clinic to examine the presence of sensory neuropathy identified by routinely applied bedside screening methods among patients with type 2 diabetes. The primary aim was to assess whether age or diabetes duration shows a stronger association with screening-detected sensory neuropathy in this population. As a secondary aim, we investigated whether commonly used diagnostic tools for neuropathy screening (128-Hz tuning fork, Semmes–Weinstein 10-g monofilament and a brief, standardized questionnaire) identify the same patient groups.

## Methods

We conducted a retrospective cohort analysis including all patients with type 2 diabetes who attended our adult internal medicine outpatient clinic in a well-defined Budapest metropolitan area between June 2017 and June 2021. The clinic provides care for approximately 20,000 adults. Patients with type 1 or type 2 diabetes were typically referred by general practitioners and were either newly diagnosed or previously treated at another institution. Diabetes was defined as any of the following: HbA1c ≥6.5% (48 mmol/mol), fasting plasma glucose ≥7.0 mmol/L (126 mg/dL), or 2-hour plasma glucose ≥11.1 mmol/L (200 mg/dL) during a standard 75-g oral glucose tolerance test. This retrospective analysis was conducted with the approval of the Regional and Institutional Committee of Science and Research Ethics of Semmelweis University (approval number: SE RKEB 8/2019).

At the first visit, a detailed medical history was obtained, including the date of diabetes diagnosis and the presence of major cardiovascular risk factors (hypertension, hyperlipidemia, smoking history and prior cardiovascular events such as acute myocardial infarction, stroke, transient ischemic attack, or peripheral vascular disease). Information on current medications was collected from GP referral letters, medical records or patient reports. Patients with active or treatment-requiring lumbar disc disease, ongoing chemotherapy, or heavy alcohol consumption (≥4 drinks/day or ≥8 drinks/week in women; ≥5 drinks/day or ≥15 drinks/week in men) were excluded from the study. Most patients presented with laboratory results performed within three months at our laboratory; in some cases, validated external laboratories (Synlab) were used. The following data were recorded: fasting plasma glucose (mmol/l), HbA1c (%), serum creatinine (µmol/l), urea (mmol/l), total cholesterol, HDL cholesterol, triglycerides (all mmol/l) and microalbuminuria (mg%). For triglyceride levels <4.5 mmol/l, LDL cholesterol was calculated using the Friedewald formula; for higher values, LDL was reported only upon special request.

Physical examinations included measurement of height (cm) and weight (kg) using calibrated equipment, blood pressure (mmHg; Omron M2 device) and heart rate (beats/min) after five minutes of rest in a seated position. Lower limb arterial circulation was assessed by palpation of the dorsalis pedis, tibialis posterior, and, if necessary, femoral arteries. Neuropathy assessments were then performed by a trained assistant.

Neuropathy testing began with inspection of the lower limbs for chronic wounds or ulcers. Pressure sensation was assessed with a 10-g Semmes–Weinstein monofilament applied to standardized sites (toe tips plantar and dorsal). A test was considered abnormal if the patient failed to perceive the monofilament on at least one toe on both feet during repeated testing. Vibration sensation was assessed using a calibrated 128-Hz tuning fork applied to the medial malleolus or, if edematous, the metatarsal head. Results were considered abnormal if vibration ceased before the tuning fork reached scale value 6 on both sides. We emphasize that neuropathy outcomes were defined as abnormal results on routinely applied bedside screening tests and were not intended to represent etiologically defined diabetic peripheral neuropathy. Patients with lower limb amputation were excluded.

Finally, patients were asked three standardized questions: (1) “Have you ever had a non-healing ulcer on any of your limbs?” (2) “Do you experience persistent pain in any lower limb at rest lasting more than 5 minutes?” and (3) “Do you feel discomfort in any lower limb at rest lasting more than 5 minutes?” Answers were recorded as “yes” or “no.” All responses were evaluated independently.

### Statistical analysis

Data obtained during the tests were arranged in a table and were made suitable for statistical processing. Data were inspected for normality visually and using normality tests. Continuous variables were presented as means ± SD and compared with a two-sample independent t-test. Categorical variables were reported as (%) and compared with a chi-square test. To compare the diagnostic value of individual neuropathy tests, Cohen’s kappa coefficient (κ) was determined, considering that this method is more robust than simple percentage agreements calculation. Kappa values were interpreted as follows: values ≤ 0 as indicating no agreement and 0.01–0.20 as none to slight, 0.21–0.40 as fair, 0.41– 0.60 as moderate, 0.61–0.80 as substantial, and 0.81–1.00 as almost perfect agreement.

The parameters independently associated with sensory neuropathy diagnosed by each method were compared using binary logistic regression models. In Model 1, age and duration of diabetes were used as indicators. Model 2 incorporated all clinically relevant variables collected (sex, smoking, hypertension, hyperlipidemia, prior myocardial infarction, prior stroke, prior peripheral arterial disease, chronic kidney disease stage III–V, family history of premature death or myocardial infarction, neuropathic symptoms, and retinopathy). Variables included in Model 2 were selected based on clinical relevance and previous studies on diabetic neuropathy, rather than on statistical significance alone. Model 3 was constructed as the most parsimonious model including only variables that statistically significantly contributed to Model 2. Multicollinearity between covariates, including age and diabetes duration, was assessed using variance inflation factors (VIF). The VIFs were all <5, suggesting no relevant collinearity. Patients with missing or incomplete neuropathy assessments or missing covariate data were excluded from the analysis. Given the retrospective design and the clinical nature of the collected variables, no data imputation was performed and analyses were conducted only in patients with complete information. Data were analyzed using IBM SPSS Statistics version 27 (IBM Corp., Armonk, NY, USA). Statistical significance was defined as p < 0.05 (two-tailed).

## Results

**Figure 1:**
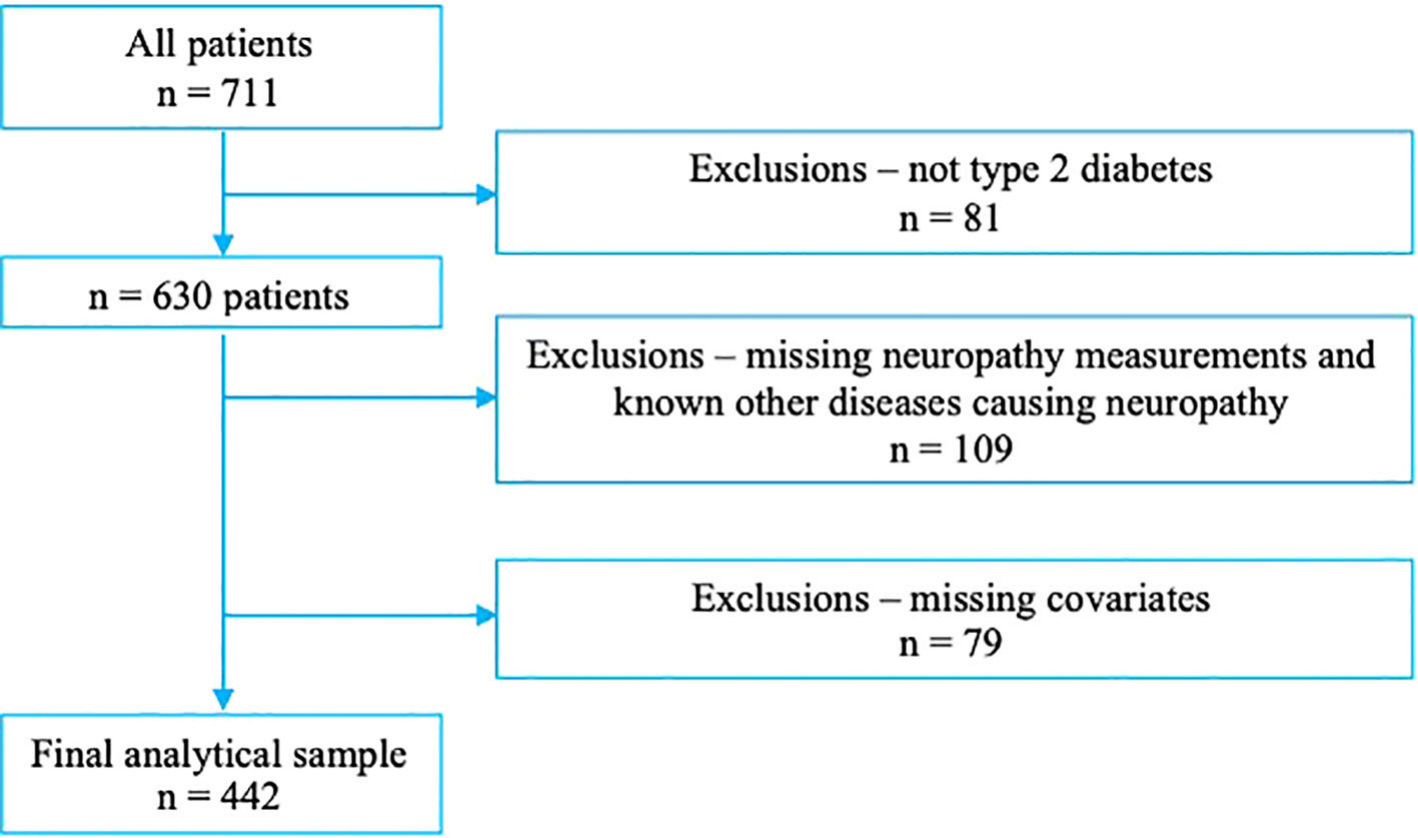
Flow chart of patient selection.

During the study period, 711 patients attended the specialist clinic, of whom 81 were not classified as having type 2 diabetes. An additional 109 patients were excluded due to missing or incomplete neuropathy assessments and 79 due to missing covariates (**Figure 1**). Ultimately, data from 442 patients were analyzed (53% male). Their mean age was 65.5 ± 11.2 years, the mean duration of diabetes was 9.6 ± 8.9 years, and the mean HbA1c was 7.8 ± 1.7%.

First, we assessed whether or not the different diagnostic methods—the calibrated tuning fork and the 10-g monofilament—identified the same patients. Interrater reliability, assessed with Cohen’s kappa, indicated only limited agreement between methods (tuning fork *vs*. monofilament κ = 0.34; 95% CI: 0.25-0.43), highlighting their restricted diagnostic overlap. This indicates that the two screening methods often identify different patient in routine outpatient practice.

Next, we examined unadjusted baseline characteristics according to the presence or absence of neuropathy as determined by each diagnostic test (**Table 1**). Given that these analyses were used only for descriptive purposes, no adjustment for multiple comparisons were made. Importantly, HbA1c values, reflecting the effectiveness of diabetes management, were nearly identical across all groups regardless of neuropathy status. Although several baseline characteristics differed between groups, age remained an independent factor associated with neuropathy in multivariable analyses. In contrast, patients with neuropathy identified by the monofilament test differed only in age, being older at baseline compared with their non-neuropathic counterparts. Symptoms of neuropathy were more frequently reported among all patients, regardless of the diagnostic tool used.

**Table 1:** Baseline participant characteristics by routine sensory neuropathy tests and their results (negative or positive for neuropathy).

	Tuning fork		p	Monofilament		p
	Negative	Positive		Negative	Positive	
n	300	142	–	322	120	–
Male	150 (50%)	81 (57%)	0.185	177 (55%)	55 (46%)	0.109
**Age (years)**	**62.8 ± 11.2**	**68.3 ± 9.9**	**<0.001**	**63.3 ± 11**	**67.9 ± 10.6**	**<0.001**
Weight (kg)	91.9 ± 20.3	93.6 ± 22.3	0.417	92.9 ± 20.8	91.1 ± 21.5	0.408
Height (cm)	167.8 ± 9.6	169.6 ± 10.1	0.073	168.7 ± 9.6	167.6 ± 10	0.311
BMI (kg/m2)	32.5 ± 6.2	32.4 ± 6.4	0.819	32.6 ± 6.2	32.3 ± 6.1	0.647
Fasting blood glucose (mmol/l)	8.6 ± 3.3	8.7 ± 3.6	0.894	8.6 ± 3.4	8.8 ± 3.4	0.66
Hba1c (%)	7.7 ± 1.6	7.8 ± 1.6	0.383	7.7 ± 1.5	7.8 ± 1.7	0.368
Creatinin (umol/l)	78.2 ± 30.2	84.5 ± 34.8	0.069	77.5 ± 23.2	87.6 ± 47.2	0.323
**Total cholesterol (mmol/l)**	**5.1 ± 1.4**	**4.8 ± 1.4**	**0.017**	5.0 ± 1.4	5.1 ± 1.3	0.692
Triglyceride (mmol/l)	2.3 ± 2.4	2.1 ± 1.2	0.929	2.2 ± 2.1	2.3 ± 2.1	0.582
Systolic blood pressure (Hgmm)	150.7 ± 20.9	150.8 ± 21	0.958	150.6 ± 20.6	151.2 ± 21.8	0.781
**Diastolic blood pressure (Hgmm)**	**88.6 ± 12.3**	**85.1 ± 11.2**	**0.002**	88.1 ± 11.9	85.9 ± 12.5	0.086
**Pulse (/min)**	**82.3 ± 13.6**	**79.7 ± 11.6**	**0.017**	82.1 ± 13.2	79.7 ± 12.3	0.080
**Diabetes duration (years)**	**8.7 ± 8.4**	**11.7 ± 9.9**	**<0.001**	9.3 ± 8.8	10.6 ± 9.5	0.065
**Smoking**	**-**	**-**	**0.007**	–	–	0.258
Never smoked	89 (29.5%)	35 (11.5%)	0	90 (29.9%)	34 (11.2%)	0
Prior smoke	42 (13.9%)	32 (10.6%)	0	50 (16.8%)	23 (7.7%)	0
Current smoking	31 (10.5%)	10 (3.4%)	0	34 (11.2%)	8 (2.7%)	0
Passive smoking	1 (0.2%)	- (0.0%)	0	1 (0.2%)	- (0.0%)	0
Hypertension	262 (87.3%)	272 (90.8%)	0.340	262 (87.3%)	275 (91.7%)	0.242
Hyperlipidaemia	197 (65.7%)	211 (70.4%)	0.331	196 (65.2%)	218 (72.5%)	0.172
**Prior myocardial infarction**	**15 (5.0%)**	**40 (13.4%)**	**0.004**	21 (7.1%)	28 (9.2%)	0.547
Prior stroke	16 (5.3%)	19 (6.3%)	0.664	14 (4.7%)	25 (8.3%)	0.164
Prior peripheral arterial disease	9 (3.0%)	11 (3.5%)	0.776	8 (2.8%)	13 (4.2%)	0.542
Chronic kidney disease, stage III-V.	1 (0.3%)	4 (1.4%)	0.243	1 (0.3%)	5 (1.7%)	0.18
Family history of premature death or myocardial infarction under age 60	24 (8.0%)	28 (9.2%)	0.714	23 (7.8%)	30 (10.0%)	0.445
**Neuropathic symptoms**			**<0.001**			**<0.001**
No neuropathic symptoms	266 (88.7%)	188 (62.7%)	0	269 (89.8%)	165 (55.0%)	
Tingle	16 (5.3%)	36 (12.0%)	0	14 (4.7%)	45 (15.0%)	
Numbness	8 (2.7%)	38 (12.7%)	0	8 (2.8%)	43 (14.2%)	
Pain	8 (2.7%)	28 (9.2%)	0	7 (2.2%)	35 (11.7%)	
Ulcer	2 (0.7%)	11 (3.5%)	0	2 (0.6%)	13 (4.2%)	
Retinopathy			0.234			0.808
No retinopathy	289 (96.3%)	275 (91.5%)	0	283 (94.4%)	287 (95.8%)	
Mild retinopathy	6 (2.0%)	13 (4.2%)	0	6 (3.1%)	5 (1.7%)	
Medium retinopathy	1 (1.3%)	2 (2.8%)	0	1 (1.9%)	2 (1.7%)	
Severe retinopathy	0 (0.3%)	2 (0.7%)	0	0 (0.3%)	1 (0.8%)	

Data are mean ± SD or n/total (%). p value was calculated by independent samples t-test or chi-square test. p < 0.05 was considered as statistically significant. BMI, body mass index; HbA1c, glycated haemoglobin A1c. Hypertension, hyperlipidaemia and diabetes mellitus were diagnosed if a patient used at least one drug for their treatment and the diagnosis was coded in their medical record by the proper ICD code (hypertension: I1xxx, hyperlipidaemia: E78xx; diabetes mellitus: E10xx, E11xx or E14xx) at least 3 times.

Bold values indicate statistically significant differences (p < 0.05).

Finally, we applied multivariable binary logistic regression in three sequential models to identify independent predictors of neuropathy (**Table 2**). Model 1 included age and diabetes duration; model 2 additionally incorporated sex, smoking status, hypertension, hyperlipidemia, prior myocardial infarction, prior stroke, prior peripheral arterial disease, chronic kidney disease (stage III–V), family history of premature death or myocardial infarction, neuropathic symptoms, and retinopathy; model 3 retained only predictors that remained significant in model 2. All models were statistically significant (p < 0.001) and showed adequate goodness of fit according to standard logistic regression diagnostics (**Table 3**). In the final reduced model, age was the only independent predictor strongly associated with neuropathy diagnosed by the tuning fork (OR 1.04; 95% CI: 1.02–1.06). This suggests that abnormal vibration perception detected by tuning fork testing is more closely related to aging than to diabetes duration in this population. Moreover, positive responses to neuropathy symptom questions reinforced the diagnostic validity of tuning fork findings. For the monofilament test, age and male sex were initially associated with positive results, but in the final model only neuropathy symptoms remained independently predictive. Accordingly, loss of pressure sensation identified by monofilament testing appeared to be independently associated with only patient-reported symptoms than with age or diabetes duration. However, associations involving rare outcomes (e.g., foot ulcers) should be interpreted with caution due to wide confidence intervals.

**Table 2:** Results of logistic regression models by routine sensory neuropathy tests in three models.

Tuning fork	
Odds ratio (95%CI)	
Model 1	
Age (years)	1.05 (1.02-1.07)
Model 2	
Age (years)	1.08 (1.04-1.12)
Neuropathic symptoms: tingle	3.01 (1.32-6.87)
Neuropathic symptoms: numbness	6.84 (2.59-18.07)
Neuropathic symptoms: pain	6.06 (2.03-18.03)
Neuropathic symptoms: ulcer	10.31 (1.71-62.11)
Model 3	
Age (years)	1.05 (1.03-1.07)
Neuropathic symptoms: tingle	2.79 (1.33-5.86)
Neuropathic symptoms: numbness	6.38 (2.65-15.36)
Neuropathic symptoms: pain	3.62 (1.41-9.27)
Neuropathic symptoms: ulcer	9.81 (1.79-53.64)

Monofilament	
Odds ratio (95%CI)	
Model 1	
Age (years)	1.04 (1.02-1.07)
Model 2	
Sex	2.46 (1.16-5.22)
Neuropathic symptoms: tingle	5.41 (2.36-12.41)
Neuropathic symptoms: numbness	9.54 (3.69-24.64)
Neuropathic symptoms: pain	7.69 (2.6-22.74)
Neuropathic symptoms: ulcer	20.42 (3.06-136.23)
Model 3	
Neuropathic symptoms: tingle	5.11 (2.44-10.7)
Neuropathic symptoms: numbness	8.3 (3.53-19.51)
Neuropathic symptoms: pain	8.57 (3.32-22.13)
Neuropathic symptoms: ulcer	12.48 (2.34-66.57)

The table shows statistically significant indicators (p < 0.05) by routine sensory neuropathy tests in three models. Model 1 used age and duration of diabetes as indicators. Model 2 used sex, smoke, hypertension, hyperlipidaemia, prior myocardial infarction, prior stroke, prior peripheral arterial disease, chronic kidney disease (stage III-V.), family history of premature death or myocardial infarction, neuropathic symptoms and retinopathy as indicators. Model 3 used those indicators which were significant in Model 2.

**Table 3:** Main characteristics of the models by routine sensory neuropathy tests.

-2 Log likelihood		Sig	Nagelkerke R^2^	Goodness of fit (Hosmer-Lemeshow)	Overall percentage
Initial	Final				
Tuning fork					
Model 1					
555.0	528.1	<0.001	0.083	0.583	69.5%
Model 2					
555.0	439.7	<0.001	0.321	0.608	76.5%
Model 3					
555.0	494.4	<0.001	0.179	0.757	72.9%
Monofilament					
Model 1					
516.9	500.9	<0.001	0.052	0.163	73.1%
Model 2					
516.9	420.3	<0.001	0.285	0.791	80.3%
Model 3					
516.9	452.7	<0.001	0.196	0.982	78.1%

Model 1 used age and duration of diabetes as indicators. Model 2 used sex, smoke, hypertension, hyperlipidaemia, prior myocardial infarction, prior stroke, prior peripheral arterial disease, chronic kidney disease (stage III-V.), family history of premature death or myocardial infarction, neuropathic symptoms and retinopathy as indicators. Model 3 used those indicators which were significant in Model 2.

## Discussion

Multiple methods are available for neuropathy screening, but clinical studies (2, 13) and physiological considerations indicate that these tests are not specific for neuropathy of diabetic origin. Consequently, diabetic neuropathy is often considered a diagnosis of exclusion (14). Since the prevalence of peripheral neuropathy increases with age (4, 15–17), it is plausible that commonly used screening methods may detect predominantly age-related rather than diabetes-associated neuropathy. In our study, neuropathy identified with the 128-Hz tuning fork was independently associated with age, whereas for the monofilament test, self-reported neuropathic symptoms showed the strongest association. Moreover, in our outpatient practice, the overlap between diagnostic methods was limited (Cohen’s κ <0.4), although all methods consistently showed a closer relationship with age rather than with diabetes duration.

DSPN is a well-established diabetic complication and an independent risk factor for mortality (2, 6). Its prevalence is approximately 22% in individuals aged 60–74 but decreases to 5% when diabetes is excluded as an etiological factor. Across various populations, neuropathy of known etiology accounts for 73–90% of cases, depending on diagnostic methods and study design (13). Although electrophysiological tests provide detailed assessments, no single method reliably distinguishes neuropathy subtypes or detects early neuronal damage. In a study, Valensi and colleagues demonstrated that among 20 electrophysiological parameters tested in patients with type 1 or type 2 diabetes, 17 correlated with diabetes duration, 9 with age, 7 with glycemic control, and only 1 with sex (18). By contrast, monofilament testing in the general population showed that neuropathy is common even without diabetes and importantly, its presence was an independent predictor of mortality (2). Neuropathy detected with monofilament is more strongly associated with diabetes in middle-aged than in older adults, while male sex and body height were also robust predictors (16). Height itself is subject to physiological fluctuations (e.g., diurnal variation, menstrual cycle) (19, 20), further complicating interpretation of data obtained by simple clinical tools.

The 128-Hz tuning fork and the 10-g Semmes–Weinstein monofilament have been used for decades because they are easy to apply, inexpensive, and widely available. However, their major limitation is that they detect only late neuronal damage (21, 22). Although both tests evaluate the same fiber type, they stimulate different receptor modalities, which may explain their limited diagnostic overlap. Moreover, small-fiber dysfunction typically precedes large-fiber damage (23, 24). As tuning fork and monofilament testing confirm only advanced large-fiber neuropathy, their negative predictive value is limited, though their positive predictive value makes them useful for annual screening and for identifying patients at risk of severe complications (25).

In routine practice, monofilament and tuning fork remain the most frequently used tools. Combining them does not substantially improve diagnostic accuracy compared with either test alone (25, 26). Nevertheless, both are recommended for diabetic populations to assess loss of protective sensation (monofilament) and vibration sense (tuning fork), which are early markers of large-fiber involvement (27). It has to be noted, that there is currently no universally accepted guideline-defined cut-off for vibration perception testing using a 128-Hz tuning fork; therefore, we applied a pragmatic dichotomization reflecting both currently utilized reimbursement rules in Hungary and the routine clinical screening practice, as well as consistent with prior diagnostic accuracy studies (28). More comprehensive approaches, such as the Michigan Neuropathy Screening Instrument (MNSI), combine questionnaires with physical examination (inspection, vibration testing, ankle reflexes) and provide a validated method to detect neuropathy (29). However, the neural pathways assessed by individual MNSI questions remain less clearly defined. In outpatient settings, MNSI has shown that diabetes duration is the strongest predictor of neuropathy severity, followed by glycemic control and age (30). Moreover, three specific questions similar to ours have been shown to provide additional prognostic information in patients with type 2 diabetes and cardiovascular disease (31). These likely capture unmeasured mechanisms, such as chronic inflammation or vascular injury. Overall, brief questionnaires alone are insufficient for neuropathy diagnosis, but they may complement objective tests.

Our results are somehow consistent with the autonomic literature: in healthy cohorts, reflex control of heart rate and heart rate variability show graded declines with advancing age, and investigators have derived age-specific reference values for standard autonomic tests (9, 10). Although contemporary guidelines typically do not mandate age-adjusted thresholds for autonomic tests (Ewing-tests), these foundational datasets support the broader concept that age exerts a generalized effect on neural performance. By analogy, the stronger association we observed between screening-detected sensory neuropathy and age - rather than diabetes duration - likely reflects this global neuro-ageing signal captured by bedside tests. Evidence shows that aging impairs peripheral nerve function and regeneration through Schwann cell dysfunction, chronic inflammation, subtle myelin changes, and increased neuronal senescence, with clinical manifestations including reduced sensory amplitudes and reflexes, and experimental evidence supporting delayed and less effective nerve repair (32–34). Therefore, screening-detected neuropathy most probably in older adults with type 2 diabetes may reflect age-related neural decline rather than diabetes-specific damage (35). However, we have to emphasize, that our results do not negate the role of diabetes duration in the development of neuropathy when more sensitive or etiologically specific diagnostic methods are applied but rather highlights the limitations of commonly used bedside screening tools in older individuals.

Our study has several limitations. As with any retrospective study, reverse causality cannot be excluded, and the cross-sectional design of the study precludes causal inference and restricts conclusions to prevalent rather than incident neuropathy. Neuropathy has a multifactorial etiology and although we excluded patients with overt non-diabetic causes when identifiable, the absence of systematic screening for all potential contributors (such as prior chemotherapy, toxin exposure, autoimmune disorders, or proven degenerative spinal disease) limits causal interpretation and may lead to residual confounding. The relatively low prevalence of retinopathy may reflect differences in patient selection and diagnostic practices and does not preclude age-related contributors to screening-detected neuropathy. Importantly, we relied on commonly used bedside screening tools and did not apply gold-standard diagnostic methods such as nerve conduction studies. While this approach reflects routine outpatient practice, it limits diagnostic specificity and the ability to distinguish diabetic neuropathy from other causes of peripheral nerve dysfunction. In addition, the results of the applied screening tests were reported to have age-dependency, which may have contributed to the strong association observed between neuropathy detection and age. However, it should be noted that age adjusted cutoff values for these screening tests are rarely applied in clinical practice. It also has to be noted, that symptoms of diabetic sensory neuropathy and peripheral arterial disease may overlap. The diagnostic overlap of tuning fork and monofilament tests was characterized by Cohen’s-kappa. The result should be interpreted with caution as although the two independent tests were completed but the same clinician performed the tests. Given that the assessor was aware of the result of the first investigation, it may have influenced the interpretation of the second one. However, this bias would result in even larger overlap between the two tests that is opposite to our finding of a limited overlap. Finally, a proportion of patients were excluded due to missing neuropathy assessments or covariates, potentially introducing selection bias. Moreover, the study population consisted exclusively of Caucasian patients, and individuals with advanced complications (e.g. prior amputation) or severe comorbidities (e.g. heart failure, advanced malignancy) were not included, limiting the generalizability of our findings to other populations and clinical settings.

Nevertheless, our study also has strengths. Testing was performed on a well-characterized population using standardized protocols, trained staff, and a relatively large sample size, which enhances the clinical relevance of our findings. Also, we maintained the focus on our principal observation that age is a stronger predictor of screening-detected sensory neuropathy than diabetes duration, providing clinically meaningful insight for everyday practice. Finally, the use of commonly applied bedside screening tools (tuning fork, monofilament, and brief symptom questions) increases the practical relevance and applicability of our results to routine outpatient care.

In summary, in routine outpatient practice, the overlap between bedside methods to detect sensory neuropathy is limited and the results are more closely associated with age rather than with diabetes duration in older adults with type 2 diabetes. Practically, incorporating age-aware interpretation (where available from normative datasets) or prioritizing modalities less sensitive to age-related change may help disentangle diabetes-specific neuropathy from background neuronal ageing in older adults. Prospective studies using gold-standard methods are needed to better distinguish diabetic from non-diabetic (primarily age-related) neuropathy and to inform appropriate therapeutic strategies.

## Data Availability

The original contributions presented in the study are included in the article/supplementary material. Further inquiries can be directed to the corresponding author.
